# Validated comprehensive RP HPLC approach for separation and quantification of solifenacin and mirabegron in the presence of their degradation products

**DOI:** 10.1038/s41598-026-39569-2

**Published:** 2026-03-13

**Authors:** Ebraam B. Kamel, Mohamed Badrawy, Israa M. Nour

**Affiliations:** Pharmaceutical Chemistry Department, Faculty of Pharmacy, Egyptian Russian University, Badr City, Cairo Egypt

**Keywords:** Mirabegron, Solifenacin, Impurities, Metabolites, HPLC, Stability-indicating, Chemistry, Drug discovery

## Abstract

A rapid and efficient High-Performance Liquid Chromatography (HPLC) method was developed and optimized for the separation and quantification of solifenacin succinate (SOL), mirabegron (MIR), solifenacin impurities A (SOL IMP A), I (SOL IMP I) and mirabegron metabolite (MIR MET) using a C_18_ column. Solifenacin succinate is a muscarinic receptor antagonist used in the treatment of overactive bladder (OAB) by reducing bladder muscle contractions, while mirabegron, a β3-adrenergic agonist, works by relaxing the bladder smooth muscle to improve storage capacity and reduce symptoms of OAB. However, the presence of impurities and metabolites, such as solifenacin impurities A, I, and E, can negatively impact the safety and efficacy of these drugs, potentially causing adverse effects such as increased toxicity, altered pharmacodynamics, or diminished therapeutic effectiveness. The method employed an isocratic elution system with a mobile phase of water containing 0.1% formic acid (solvent A) and acetonitrile with 0.1% formic acid (solvent B), using a C_18_ column (4.6 mm × 100 mm, 3 μm) at a flow rate of 1.2 mL/min and a column temperature of 30 °C. UV detection at 220 nm allowed for the efficient separation of all analytes within 10.5 min, with excellent resolution between solifenacin succinate, mirabegron, their metabolites, and impurities. The linearity range for solifenacin succinate and mirabegron was 1–100 µg/mL, and solifenacin impurities A, I, and E, and mirabegron metabolite exhibited linearity ranges of 0.1–10 and 0.1–50 µg/mL, respectively. Validation studies confirmed good precision (RSD < 2%) and accuracy, making this method suitable for routine quality control and pharmacokinetic studies. This validated HPLC method offers a reliable tool for monitoring the purity of solifenacin succinate and mirabegron, ensuring their therapeutic efficacy and safety by quantifying both the active compounds and potentially harmful impurities or metabolites.

**Supplementary Information:**

The online version contains supplementary material available at 10.1038/s41598-026-39569-2.

## Introduction

High-Performance Liquid Chromatography (HPLC) has become an essential tool in pharmaceutical analysis due to its high sensitivity, precision, and versatility in separating, quantifying, and characterizing active pharmaceutical ingredients (APIs) and their impurities. Solifenacin succinate and mirabegron are two widely prescribed drugs for the treatment of overactive bladder (OAB) syndrome, but their quality and safety can be compromised by the presence of impurities and metabolites. Monitoring the levels of these compounds, as well as impurities such as solifenacin impurities A, I, and E, is critical for ensuring the therapeutic efficacy and safety of these drugs.

Solifenacin Succinate (SOL), its chemical name is (R)-N-[(S)-1-phenylpropan-2-yl]-1,3-dihydro-2 H-imidazo[4,5-b]quinolin-2-one succinate, and its molecular weight is 453.57 g/mol. SOL is listed in the United States Pharmacopeia (USP)^1^ and the European Pharmacopoeia (EP)^2^, which ensures its purity and quality standards.

SOL is a muscarinic receptor antagonist that selectively inhibits the M3 muscarinic receptor in the bladder, which is responsible for contraction of the detrusor muscle. By blocking this receptor, solifenacin reduces involuntary bladder contractions and increases bladder capacity, thereby alleviating the symptoms of OAB such as urinary urgency, frequency, and incontinence. It has a high selectivity for M3 receptors over other muscarinic subtypes, which reduces side effects like dry mouth and constipation^3^.

SOL is prone to several impurities, including Impurity A, Impurity I, and Impurity E, which may arise from the synthesis process. These impurities could potentially alter the drug’s pharmacokinetics and pharmacodynamics. Impurity A has been linked to increased toxicity in preclinical studies, while Impurity I may affect the receptor-binding affinity of the drug, leading to suboptimal therapeutic effects. Impurity E may cause adverse gastrointestinal effects, potentially reducing patient compliance.

An extensive review was done in the literature to cite the determination of SOL either alone or in combination and also with its degradation products and/or impurities in different matrices. Several analytical methods like HPLC with different detectors were published^4–16^, HPTLC^17–19^, and spectroscopic methods^20–26^ were published.

Mirabegron (MIR), its chemical name is (S)-N-[2-(2-amino-3,5-dichlorophenyl)-1,3-thiazol-4-yl]-N’-cyclopropylmethanamide, and molecular weight is 413.90 g/mol. MIR is listed in the United States Pharmacopeia (USP)^1^.

 MIR is a selective β3-adrenergic agonist. It works by binding to β3-adrenergic receptors in the bladder, leading to relaxation of the detrusor muscle and an increase in bladder capacity. This mechanism contrasts with that of muscarinic antagonists, offering a complementary approach to treating OAB. By stimulating the β3-adrenergic receptor, MIR helps to improve symptoms of OAB without causing the dry mouth and constipation commonly associated with muscarinic antagonists^27,28^.

Similar to SOL, MIR can contain impurities, although fewer studies have been published on these in the literature. Impurities in MIR may affect its pharmacokinetics and could lead to altered drug metabolism, potentially reducing the drug’s efficacy or causing undesirable side effects. The structural analogs and degradation products, including metabolites of MIR, may possess weaker or even toxic pharmacological activity, emphasizing the need for thorough impurity profiling^29^.

An extensive review was done in the literature to cite the determination of MIR either alone or in combination and also with its degradation products and/or impurities in different matrices, several analytical methods, like HPLC with different detectors were published^30–34^, HPTLC^35,36^, spectroscopic^35,37–40^, and also electrochemical^41,42^ methods were published.

The optimized HPLC method described in this study provides an efficient, reliable, and rapid approach for the separation and quantification of SOL, MIR, their metabolites, and/or impurities. By combining pharmacological insights, analytical efficiency, and regulatory compliance, this method contributes significantly to improving the quality control of pharmaceutical products containing these drugs. Furthermore, it highlights the importance of impurity profiling and metabolite monitoring in ensuring the safety, efficacy, and therapeutic reliability of medications used to treat overactive bladder syndrome.

According to the literature review, many analytical methods were published for the determination of SOL and MIR in different matrices^12,40,43–47^.

The method presented in this study offers a significant advancement over previously published methods in terms of separation efficiency, analysis time, and linearity. By using a shorter C_18_ column (100 mm) with a small particle size (3 μm) and optimizing the gradient elution profile, this method reduces the total run time to 10.5 min, which is a considerable improvement over previous methods that typically required 30 min or longer. The flow rate (1.2 mL/min) and elevated column temperature (30 °C) further contribute to faster analysis without compromising resolution. The UV detection at 220 nm provides excellent sensitivity for SOL, MIR, and their metabolites/impurities, ensuring the detection of both major and trace components in pharmaceutical formulations. This method is particularly advantageous for routine quality control and pharmacokinetic studies, as it offers robustness, precision, and the ability to detect even low levels of impurities and metabolites. Additionally, this method is particularly useful for regulatory compliance in the pharmaceutical industry, ensuring that SOL and MIR formulations meet established pharmacopoeial standards.

## Experimental

### Instruments

An Agilent 1100 series HPLC system was employed, consisting of a quaternary pump (model G1311A), diode array detector (model G1314A), degasser (model G1322A), and an automatic injector with a 1 µL sample loop. Separation was achieved on a Zorbax Eclipse Plus C_18_ column (4.6 × 100 mm, 3 μm, USA) maintained at 30 °C.

Additional equipment included a Jenway digital ion analyzer (model 3505, Felsted, Dunmow, Essex, UK) with a glass pH electrode (Jenway, UK), and an analytical balance (Precisa125A, Switzerland). Sample handling and preparation were facilitated using a vortex mixer (IVM-300P, Taiwan), a bench-top centrifuge with a maximum speed of 6000 rpm (Hunan, China), and a rotary evaporator (Scilogex RE 100-pro, USA).

Filtration of samples and mobile phase was performed using 0.45 μm MS^®^ nylon membrane filters (USA) and 0.22 μm MS^®^ disposable syringe filters (USA), respectively. A Cleanwise^®^ sonicator and a Centurion Scientific Ltd^®^ refrigerated centrifuge were used for the preparation of standard and pharmaceutical dosage form sample solutions.

### Reagents and materials

#### Pure samples

SOL and MIR were graciously provided by Marcryl Pharmaceuticals (Cairo, Egypt) with certified purity of 99.89% and 99.88%, respectively.

#### Pharmaceutical preparation

Sofenacin plus 5/50 tablet (Batch No. 23434222) manufactured by Marcyrl Pharmaceutical Industries, purchased from a local pharmacy.

#### Degraded samples

All the degradation substances, namely, SOL IMP A, SOL IMP I, SOL IMP E and MIR MET have been prepared as subsequently shown.

#### Chemicals and reagents

HPLC-grade chemicals and solvents with sodium hydroxide, hydrochloric acid, and hydrogen peroxide (El-Nasr company, Cairo, Egypt) were employed. Formic acid and acetonitrile with HPLC-grade (Fisher Scientific, UK), and double-distilled water were also used.

### SOL and MIR stress stability studies

Stress testing was conducted in accordance with ICH guidelines^48^ under acidic, basic, oxidative, photolytic, and thermal conditions. For acidic hydrolysis, 100 mg of each drug was dissolved separately in aqueous HCl solutions (0.5–3 M) and refluxed in a round-bottom flask at 100 °C for 12 h. Basic hydrolysis was performed in the same manner using aqueous NaOH solutions of equivalent molarity. For oxidative degradation, 10 mg of each drug was refluxed individually in aqueous H₂O₂ solutions (0.5–10%, w/v) for 12 h at 100 °C. Photolytic stability was evaluated by exposing the powdered drug to UV light (254 nm) for 12 h at a distance of 10 cm from the light source. The drug was evenly dispersed as a thin layer in a Petri dish during irradiation. Thermal stability was assessed by placing glass ampoules containing the drug in a thermostatically controlled oven at 50, 60, and 100 °C for 12 h. In all cases, aliquots were withdrawn at predetermined intervals, neutralized, diluted to appropriate concentrations, and analyzed for the extent of degradation using the proposed methods. The objective of the forced degradation studies was not to establish degradation kinetics or perform a full mass balance evaluation, but rather to generate representative degradation products under stressed conditions to support the development of a stability-indicating chromatographic method. Therefore, the applied stress conditions were designed to ensure sufficient degradation and enable clear chromatographic separation and identification of degradation products relevant to method selectivity.

### Preparation of degradation products

#### SOL hydrolytic degradation products (SOL IMP A and SOL IMP E)

A 100 mg portion of SOL was refluxed in a round-bottom flask with 100 mL of 3 M HCl or 3 M NaOH at 100 °C for 12 h. These conditions were selected to ensure complete degradation and reliable identification of the major degradation products in accordance with ICH recommendations. The resulting solution was neutralized using a pre-calculated volume of 5 M NaOH (for acidic hydrolysis) or 5 M HCl (for basic hydrolysis). The neutralized mixture was evaporated to dryness, and the residue was purified in methanol. Structural elucidation of the purified degradation products was performed using infrared (IR) and mass spectrometry (MS). Preparative thin-layer chromatography (TLC) was employed to separate individual degradation products, which were then reserved for subsequent method development. While 12 h reflux at 100 °C in strong acid or base ensures complete degradation, these harsh conditions may produce multiple secondary degradation products, potentially complicating isolation and characterization. Optimization of time and concentration could improve selectivity for primary impurities (IMP A and IMP E). While SOL IMP E was characterized and also separated, it cannot complete the method development stage because it is UV inactive.

#### SOL oxidative degradation product (SOL IMP I)

10 mg of SOL were refluxed in a round-bottom flask with 100 mL of 10% aqueous H₂O₂ for 15 h at 100 °C. These conditions were selected to ensure complete degradation and reliable identification of the major degradation products in accordance with ICH recommendations. The solution pH was adjusted to neutrality, and excess hydrogen peroxide was removed via evaporation. The dried residue was purified with methanol, and the isolated solid was characterized by IR and MS. The extended exposure (15 h) to high-concentration H₂O₂ at elevated temperatures may risk over-oxidation and non-specific degradation. Using milder oxidative conditions or shorter reaction times could enhance selectivity for the desired impurity (IMP I).

#### MIR hydrolytic degradation product (MIR MET)

50 mg of pure MIR were refluxed with 50 mL of 1 N HCl at 100 °C for 9 h, or with 1 N NaOH at 100 °C for 7.5 h. After degradation, the solutions were neutralized and evaporated to dryness. The solid residues were extracted with methanol and again evaporated to dryness. The final residues were characterized by IR and MS. Although the acidic and basic conditions employed are sufficient for inducing hydrolysis, the relatively long reflux periods could promote degradation beyond the targeted MIR MET product. Preliminary kinetic studies could help determine the minimal exposure time needed to selectively produce the desired degradation product.

### Standard solutions

Standard stock solutions of SOL, MIR, and their potential degradation products were prepared in methanol at a concentration of 1 mg/mL. Working solutions (100 µg/mL) were prepared by appropriate dilution of the stock solutions with the mobile phase.

### Procedures

#### Chromatographic conditions

Chromatographic separation was achieved on a C_18_ Zorbax^®^ SB column (4.6 × 100 mm, 3 μm) using an isocratic elution system. The mobile phase consisted of 0.1% formic acid in water (A) and 0.1% formic acid in acetonitrile (B) in a ratio of 10:90 (v/v), delivered at a flow rate of 1.2 mL/min. Both mobile-phase components were filtered through a 0.45 μm Millipore membrane filter and degassed in an ultrasonic bath for 15 min prior to use. Detection was performed at 220 nm. Samples were also filtered through a 0.45 μm membrane filter before injection. A 20 µL aliquot was injected in triplicate using an autosampler for each analysis.

#### Linearity

Linearity was assessed by serial dilution of the analytes from the corresponding stock and working standard solutions. The linearity ranges were 1–100 µg/mL for SOL and MIR, 0.1–10 µg/mL for SOL IMP A and SOL IMP I, and 0.1–50 µg/mL for MIR MET. Each prepared solution was injected in triplicate under the optimized chromatographic conditions. Calibration curves were constructed by plotting the mean peak areas against the corresponding concentrations, demonstrating the linear response of the method within the specified ranges.

## Results and discussion

A comparative evaluation of the proposed RP-HPLC method with previously reported analytical methods for the determination of SOL and MIR is presented in Table S1 (supplementary). The comparison was performed based on key validation parameters, including linearity ranges, limits of detection (LOD), limits of quantification (LOQ), and total analysis time. As shown in Table S1 (supplementary), several reported methods focus primarily on the simultaneous determination of the two drugs without addressing impurity or degradation product profiling, while others rely on non-chromatographic techniques that lack adequate selectivity for stability-indicating purposes.

In contrast, the proposed method demonstrates superior analytical performance by enabling the simultaneous separation and quantification of SOL, MIR, their official impurities (IMP A and IMP I), and the major degradation/metabolite product of MIR within a single chromatographic run. The method exhibits wider linearity ranges and lower LOD and LOQ values compared to most reported RP-HPLC methods, while achieving a significantly shorter run time (10.5 min). These features, combined with the use of conventional RP-HPLC instrumentation, make the proposed method particularly suitable for routine quality control and stability studies in pharmaceutical laboratories.

### Optimization of HPLC conditions

The C_18_ stationary phase was selected due to its proven ability to provide strong retention and effective separation for non-polar to moderately polar compounds such as SOL, MIR, and their degradation products. Incorporating 0.1% formic acid in both aqueous and organic phases enhances chromatographic performance by suppressing analyte ionization, thus improving peak symmetry and reproducibility. The high proportion of acetonitrile (90%) in the mobile phase facilitates the elution of more hydrophobic components, reducing analysis time while maintaining resolution. The column temperature was maintained at 30 °C to ensure reproducible retention times and consistent separation. While higher column temperatures can lower mobile phase viscosity and enhance peak shape, excessive heating could lead to solvent volatility issues or potential analyte degradation, particularly for thermolabile degradation products.

A detection wavelength of 220 nm was chosen for its high sensitivity toward SOL, MIR, and their related impurities, as these compounds exhibit strong UV absorbance near this region. This ensures adequate detection sensitivity even at low impurity levels.

**Fig. 1:**
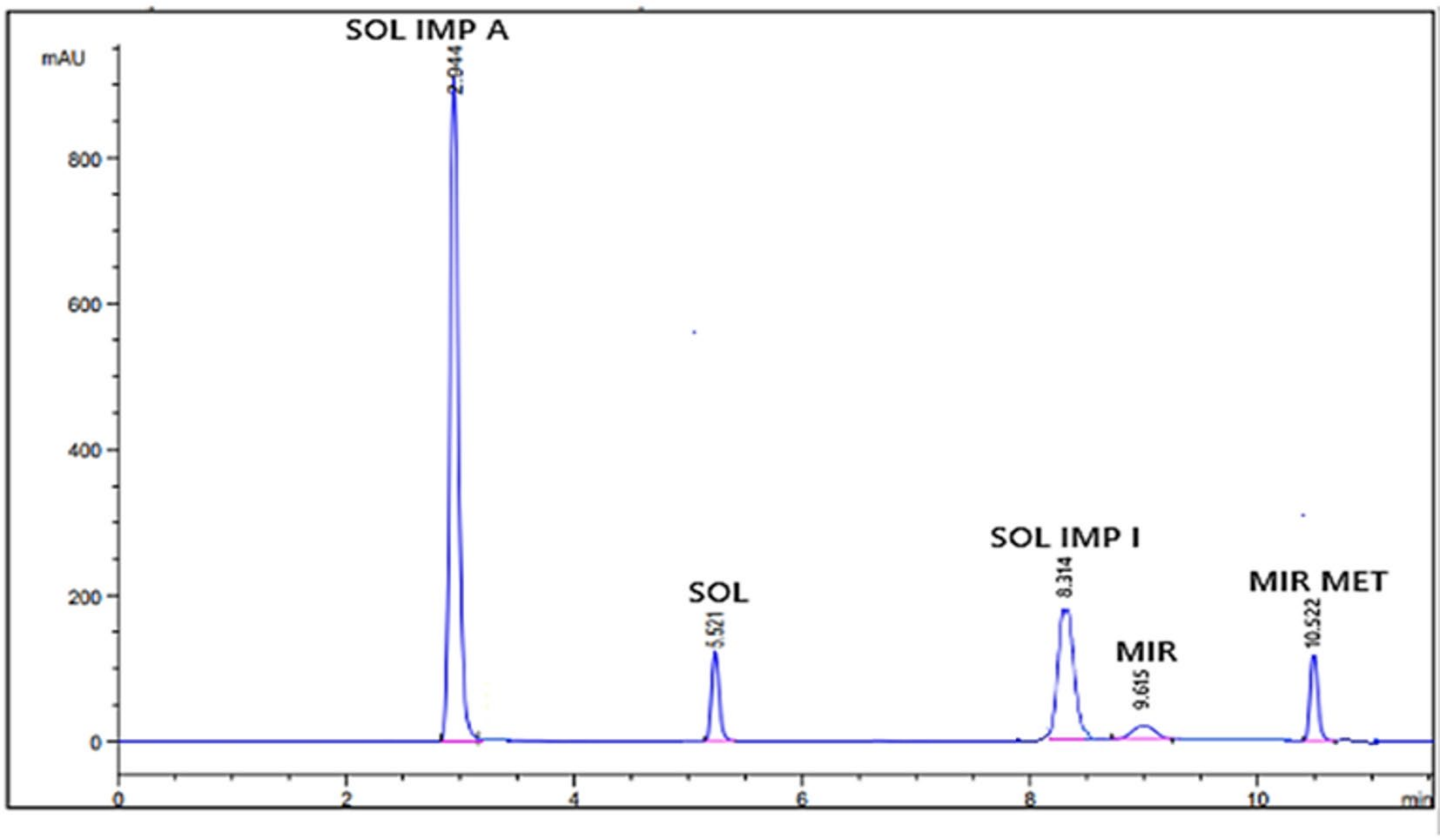
HPLC separation of SOL, MIR, SOL IMP A, SOL IMP I, and MIT MET using a mobile phase of water containing 0.1% formic acid (solvent A) and acetonitrile with 0.1% formic acid (solvent B) at a flow rate of 1.2 mL/min with a UV detection at 220 nm.

Under the optimized chromatographic conditions, the analytes were well resolved, with retention times of 5.5 min (SOL), 9.6 min (MIR), 2.9 min (SOL IMP A), 8.3 min (SOL IMP I), and 10.5 min (MIR MET) as seen in Fig. 1. System suitability parameters, including theoretical plate count, resolution, and tailing factor, were within the acceptable limits defined by USP guidelines^1^ (Table 1), confirming the method’s robustness and efficiency.

**Table 1:** System suitability parameters of the proposed HPLC–DAD method for determination of SOL and MIR along with their possible degradation products and/or metabolite.

Method	Parameter	SOL IMP A	SOL				SOL IMP I		MIR		MIR MET
	Retention time t_r_(min)	2.9	5.2				8.3		9.6		10.5
HPLC	Capacity factor (k^0^)	2.56	3.83				5.78		6.83		7.63
	Selectivity(α)		1.49	1.18		1.72		1.18		1.11	
	Resolution (Rs)		6.54	4.24		3.57		2.78		3.84	
	Tailing factor(T)	1.65	1.12				1.09		1.22		1.54
	Column efficiency (N)	3567	2543				5668		8378		12,567
	Height equivalent to theoretical plate(cm/plate)	0.0028	0.0039				0.0017		0.0011		0.00079

### Structure elucidation of possible degradation products

The chemical stability of the two drugs, SOL and MIR, was investigated under different stress conditions as per ICH guidelines^48^. The stability studies were monitored periodically by using HPTLC by using ethyl acetate: toluene: ethanol: ammonia (5: 2: 3: 0.4, by volume) as a mobile phase. Although mass balance was not quantitatively evaluated in this study, chromatographic analysis confirmed complete resolution of all detectable degradation products with no evidence of co-elution or unresolved peaks, supporting the selectivity and reliability of the proposed method.

#### Characterization of SOL with its possible degradation products

SOL underwent complete degradation upon refluxing at 100 °C with 3 M NaOH or 3 M HCl for 12 h, as well as upon oxidative treatment with 10% H₂O₂ under similar conditions. The completion of basic, acidic, and oxidative degradation was confirmed by the disappearance of the HPTLC spot at Rf = 0.42, corresponding to the intact drug. In contrast, SOL exhibited high thermal and photolytic stability, with no significant degradation observed under these conditions.

**Fig. 2:**
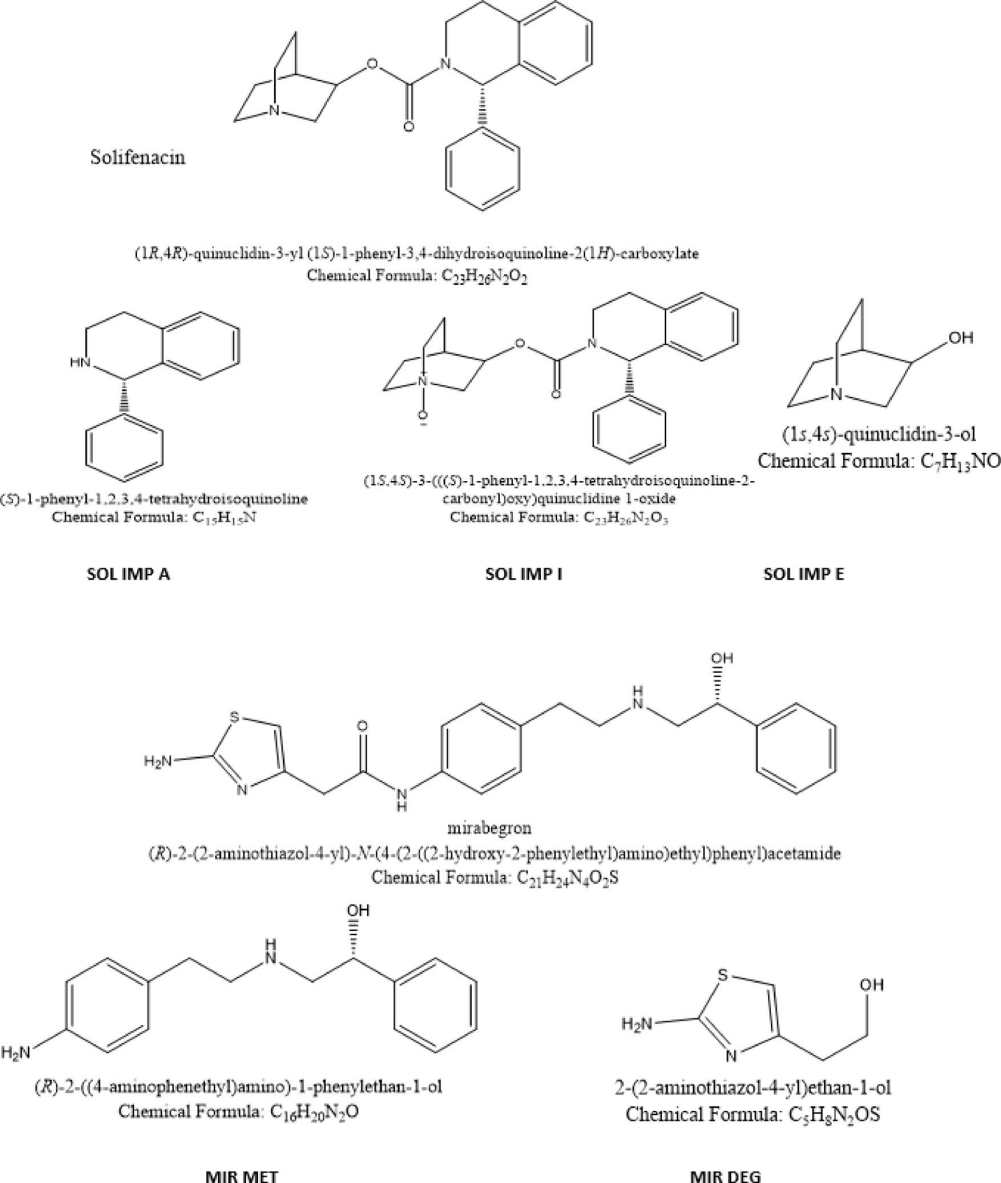
Chemical structures of SOL, MIR, SOL IMP A, SOL IMP I, SOL IMP E, MIT MET, and MIR DEG.

The molecule’s carbamate functional group was particularly susceptible to acid- and base-catalyzed hydrolysis, producing the official impurities SOL-imp-A and SOL-imp-E (Fig. 2). As SOL IMP-E lacks UV chromophores, it was visualized using iodine vapor exposure for 5 min suitable for qualitative identification but not for quantitative analysis^49^.

Oxidative degradation proceeded via a nucleophilic pathway, generating SOL-imp-I through N-oxide formation at the tertiary amine of the quinuclidine ring (Fig. 2). This reaction is facilitated by the availability of the nitrogen’s lone pair for oxygen binding, in contrast to the nitrogen in the tetrahydroisoquinoline ring, whose lone pair is delocalized via resonance with the adjacent carbonyl group, rendering it less reactive toward oxidation^50^.

All degradation products were characterized by IR and mass spectrometry. The mass spectra revealed molecular ion peaks at m/z 362 for SOL and m/z 379 for SOL-imp-I (Figs. S1–S2). Acidic/basic hydrolysis yielded SOL-imp-A (m/z 210) and SOL-imp-E (m/z 128) (Fig. S3). The IR spectrum of the hydrolysis products showed loss of the carbamate C = O band at 1720 cm⁻¹ and the appearance of a broad N–H/O–H band at 3400 cm⁻¹, consistent with carbamate cleavage. In contrast, the IR spectrum of SOL-imp-I retained the carbamate C = O absorption^51^, with no major spectral shifts (Figs. S4–S6).

While the degradation pathways and product identification were clearly established, the study could be strengthened by quantifying the degradation kinetics under each stress condition to better understand relative stability. Additionally, although iodine vapor provided a qualitative visualization for SOL-imp-E, the lack of UV activity highlights a limitation in HPTLC quantification alternative detection techniques (e.g., derivatization or LC-MS) would improve sensitivity and specificity. Finally, the observed thermal and photolytic stability warrants further exploration, as prolonged exposure times or higher energy light sources might reveal additional minor degradation pathways relevant to long-term storage.

#### Characterization of MIR with its possible degradation products

To ensure complete degradation of MIR and isolate its final degradation product, experiments were conducted using 1 N HCl and 1 N NaOH under reflux. The degradation process was continuously monitored using TLC. The characteristic MIR spot completely disappeared after 9 h in acidic conditions and 7.5 h in alkaline conditions, indicating full hydrolysis. In both cases, the TLC chromatogram showed a single degradation spot with identical Rf values for acidic and basic hydrolysis products. Visualization with iodine vapor confirmed the absence of any additional degradation products.

The isolated degradation product, identified as MIR MET (Fig. 2), was structurally elucidated and confirmed via mass spectrometry and IR spectroscopy. The MS spectrum of intact MIR exhibited a molecular ion peak at m/z 397 (Fig. S7), while MET displayed a parent ion peak at m/z 257, corresponding to its proposed molecular weight (Fig. S8). The IR spectrum of MIR revealed a carbonyl (C = O) sharp peak at 1651 cm⁻¹ and distinctive thiazole ring vibrations in the range of 600–1400 cm⁻¹ (Fig. S9). These characteristic thiazole-related absorptions were absent in the IR spectrum of MIR MET (Fig. S10), confirming thiazole ring cleavage during degradation.

The absence of detectable thiazole-containing fragments in the final degradation mixture suggests possible photodegradation of 2-(2-amino-1,3-thiazol-4-yl)acetic acid, potentially involving ring-opening and complete structural breakdown under visible light. Interestingly, MIR MET has been reported as a major in vivo metabolite of MIR in humans, with amide hydrolysis identified as the predominant metabolic pathway. This correlation underscores the pharmacological and toxicological relevance of our findings and reinforces the value of the developed chromatographic separation method, which enables the simultaneous determination of MIR and its hydrolytic degradation product.

While the study conclusively identifies MIR MET as the sole stable degradation product under acidic and alkaline conditions, the absence of intermediate products raises the question of whether transient species may have formed but degraded rapidly under the experimental conditions. Real-time LC-MS monitoring during hydrolysis could provide deeper insight into the reaction mechanism. Furthermore, the proposed photodegradation pathway of the thiazole moiety remains speculative^52,53^; targeted photolysis experiments and high-resolution MS analysis could confirm the identity of any minor by-products. Finally, since MET is a known human metabolite, stability and safety profiling of MIR MET itself could enhance the regulatory significance of the current work^54^.

### Method validation

The proposed chromatographic methods were validated in accordance with the International Council for Harmonisation (ICH) Q2(R1) guidelines^55^, covering the parameters of linearity, range, accuracy, precision, limits of detection (LOD) and quantification (LOQ), specificity, and robustness. The method demonstrates strong compliance with ICH Q2(R1) requirements, with all validation parameters meeting acceptance criteria. However, further evaluation under transferability conditions (e.g., between laboratories or using different column batches) could strengthen the robustness claim. Additionally, given the high sensitivity, the method could potentially be applied to pharmacokinetic or bioequivalence studies, provided matrix effects are thoroughly investigated. Validation was performed separately for each analyte, including all impurities and the degradation product.

#### Linearity and range

Calibration curves were constructed by plotting the mean integrated peak areas against the corresponding analyte concentrations, followed by computation of the regression equations. As summarized in Table 2, both SOL and MIR, as well as their degradation products, demonstrated excellent linearity within the tested ranges. The high correlation coefficients (*r* > 0.999) confirm the suitability of the proposed methods for quantitative analysis across a wide concentration range.

**Table 2:** Regression and validation parameters of the proposed HPLC–DAD for determination of SOL and MIR along with their possible degradation products and/or metabolite.

Method parameter	SOL IMP A	SOL		SOL IMP I	MIR	MIR MET
Range (µg/mL)	0.1–10	1-100		0.1–10	1-100	0.1–50
Slope	177.23	95.29		245.05	62.83	91.45
Intercept	-12.81	16.34		-1.74089	61.45	62.435
Correlation coefficient (r)	0.9999	0.9999		0.9999	0.9999	0.9996
Accuracy (mean ± SD)	100.85± 0.643	99.23± 0.687		101.35± 1.238	99.82± 1.036	100.88± 1.045
Robustness (RSD%)	--	1.834		--	1.347	--
LOD (μg/mL)	0.029	0.295		0.227	0.329	0.031
LOQ (μg/mL)	0.088	0.896		0.032	0.997	0.095
Repeatability (%RSD)	0.974	0.355		0.098	1.073	1.167
Intermediate precision (%RSD)	0.874	0.985		1.173	1.427	1.634
Specificity (mean ± SD)	100.22± 0.639	100.36± 0.736		98.61± 0.827	99.73± 0.825	99.93± 0.427

#### Accuracy

Accuracy was assessed by analyzing two different concentrations of pure SOL and MIR under the optimized chromatographic conditions. The concentrations were calculated using the regression equations obtained for each drug. The mean percentage recoveries (Table 2) were within the acceptable range (98–102%), indicating the high trueness of the method and confirming that the procedure is free from significant systematic error.

#### Precision

Intra-day and inter-day precisions were evaluated by analyzing three selected concentrations of each drug in triplicate, both within the same day and over three consecutive days. The calculated relative standard deviations (RSD%) were consistently below 2% (Table 2), reflecting excellent repeatability and intermediate precision, and confirming that the method produces reproducible results under normal operating conditions.

#### Specificity

Specificity was confirmed through the complete chromatographic resolution of SOL, MIR, and their degradation products without interference from formulation excipients or other potential impurities. Peak purity analysis further confirmed the absence of co-eluting species, and chromatograms obtained from placebo samples revealed no detectable peaks at the retention times of the analytes of interest. This demonstrates the method’s capability to selectively quantify the target analytes in the presence of potential interferences.

#### Limits of detection and quantification

LOD and LOQ values were calculated according to ICH guidelines using the slope of the calibration curves and the residual standard deviation (σ) of the response. The low LOD and LOQ values for both drugs and impurities (Table 2) indicate high sensitivity, enabling trace-level detection well below pharmacopeial impurity thresholds. This feature is particularly advantageous for stability studies and quality control of impurities.

#### Robustness

Robustness was examined by introducing small, deliberate variations in chromatographic parameters, including detection wavelength (± 1 nm), flow rate (± 0.1 mL/min), and buffer pH (± 0.1 units). These variations produced no significant change in peak area, retention time, or resolution. All recovery values remained within 98–102%, and the pooled RSD% did not exceed 2%, confirming the reliability and stability of the method under slight operational fluctuations.

### Analysis of the pharmaceutical formulation (sofenacin plus^®^ tablet)

The applicability of the proposed methods was verified by analyzing Sofenacin plus^®^ tablets, a co-formulated dosage form containing SOL and MIR in disparate ratios. A single-step methanol extraction was employed, which proved efficient for both analytes without requiring additional clean-up procedures. Chromatographic analysis confirmed the absence of interference from excipients, as no extraneous peaks were detected at the retention times of the target compounds.

To further validate the method in the presence of the formulation matrix, a standard addition technique was performed. The recovery results, summarized in Table 3, demonstrated values within the acceptable range (98–102%), supporting the accuracy of the method when applied to real pharmaceutical samples.

**Table 3:** Determination of SOL and MIR by the suggested chromatographic technique in Sofenacin plus^®^, and application of standard addition technique.

Platforms Parameters	HPLC	
	SOL	MIR
Sofenacin plus^®^^a^ Found % ± SD	100.87 ± 1.34	100.62 ± 1.32
Standard addition Pure found% ± SD^b^	99.55 ± 1.22	100.67 ± 0.43

^a^Sofenacin plus^®^ labeled to contain 5 mg of SOL and 50 mg of MIR; batch number (23434222).

^b^Mean of five determinations.

The successful application to a fixed-dose combination highlights the selectivity and robustness of the proposed approach, particularly in handling large ratio disparities between active ingredients. However, the study could be strengthened by including forced degradation of the final tablet formulation to assess potential interference from degradation products formed under stress conditions. This would further establish the stability-indicating capability of the method in a real-world setting.

### Statistical analysis

A comparative statistical analysis was performed between the results obtained for the analysis of SOL and MIR in the pharmaceutical dosage form by the proposed method and those attained previously using spectrofluorometer^45^. The calculated t and F values are lower than the tabulated values, indicating no statistically significant difference between the suggested and reported methods in terms of both accuracy and precision (Table 4).

**Table 4:** Statistical comparison of the results obtained for SOL and MIR by the proposed HPLC method and reported method.

Parameters	Proposed methods		Reported method^45a^	Reported method^45a^
	SOL	MIR	SOL	MIR
Mean	100.87	100.62	99.51	101.30
SD	1.34	1.32	1.22	0.55
Variance	1.796	1.742	1.488	0.303
*t*-test	1.30 (2.78)	1.21 (2.78)	–	–
F-value	1.21 (19.00)	5.76 (19.00)	–	–

^a^HPLC method with a mobile phase consisted of 0.1% OPA and 25:75% vol/vol methanol along with TEA, pH 4.2, flow rate of 0.7 mL/min, and detected at 231 nm using a UV detector.

## Conclusion

The developed HPLC method offers a robust, precise, and reproducible approach for the simultaneous separation and quantification of SOL and MIR with their possible degradation products using a C_18_ column. The optimized chromatographic conditions including isocratic elution, carefully selected mobile phase composition, and UV detection at 220 nm achieved excellent resolution, peak symmetry, and sensitivity, making the method suitable for routine quality control and stability-indicating analysis.

While the method demonstrated high selectivity and reliability, minor optimization of the gradient profile or mobile phase composition may be required when applied to complex or novel sample matrices to ensure consistent performance. Nevertheless, the proposed procedure provides a robust analytical platform for routine pharmaceutical analysis, impurity profiling, and potential application in bioanalytical studies, provided it is appropriately validated.

## Supplementary Information

Below is the link to the electronic supplementary material.

Supplementary Material 1

## Data Availability

The datasets used and/or analyzed during the current study are available from the corresponding author on reasonable request.
