# Supplementary material for: Validated comprehensive RP HPLC approach for separation and quantification of solifenacin and mirabegron in the presence of their degradation products
Source: Sci Rep. 2026 Mar 13;16:9341. doi: 10.1038/s41598-026-39569-2 (PMC13002987; doi:10.1038/s41598-026-39569-2)
Supplement: Supplementary file 1 — Supplementary Material 1 [file 41598_2026_39569_MOESM1_ESM.docx]

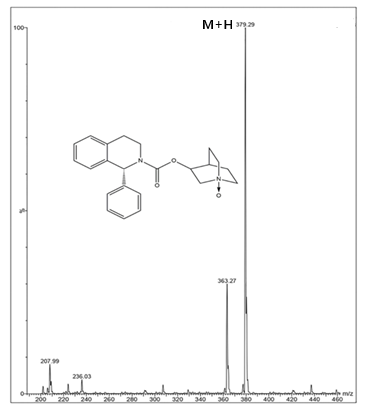

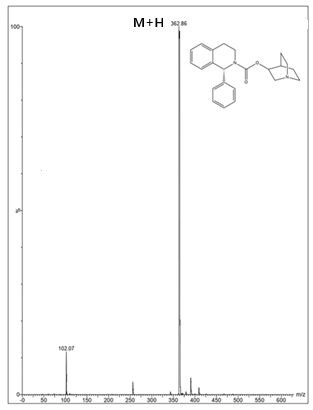
Fig S1 MS spectrum of SOL Fig S2 MS spectrum of SOL IMP I


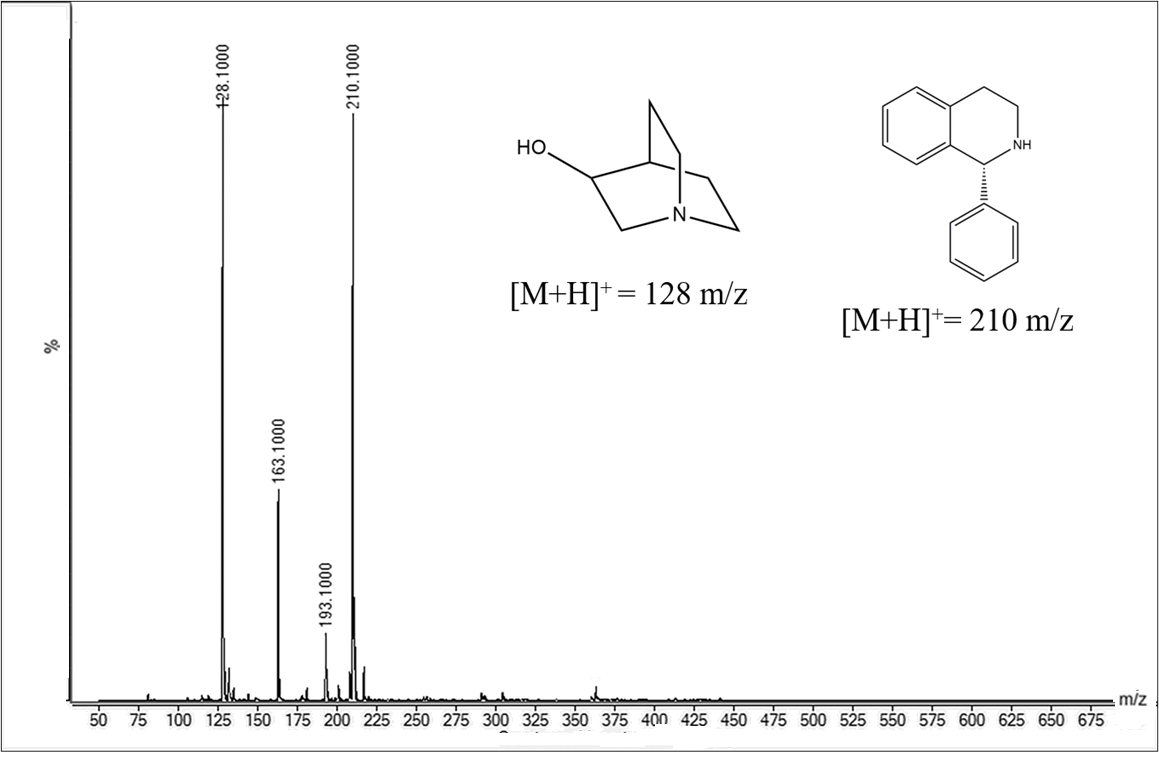


Fig S3 MS spectrum of SOL IMP A and SOL IMP E

Fig S4 IR spectrum of SOL
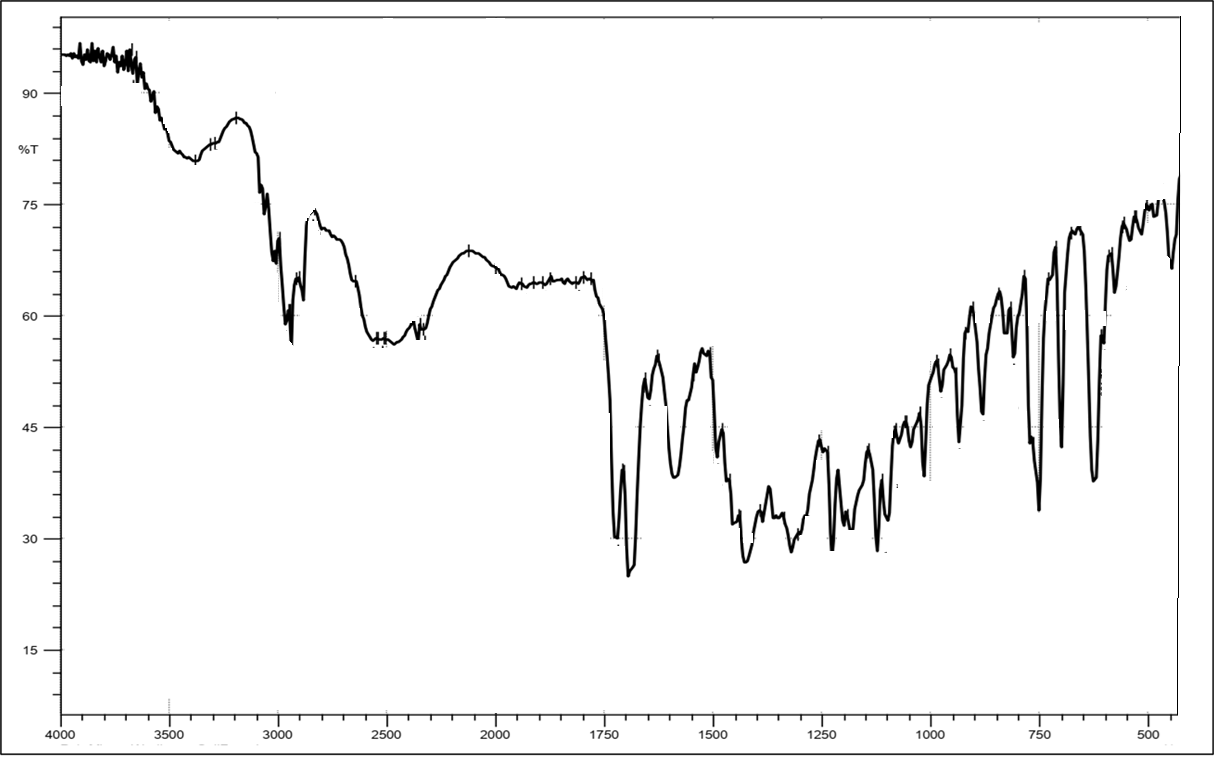


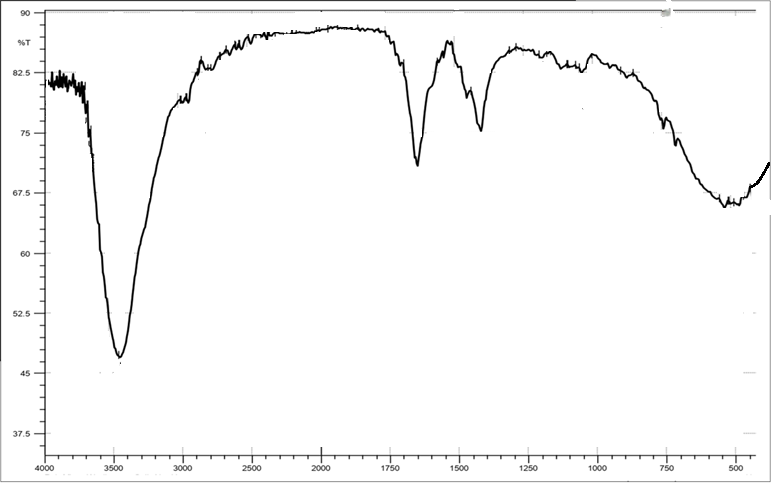


Fig S5 IR spectrum of SOL IMP I


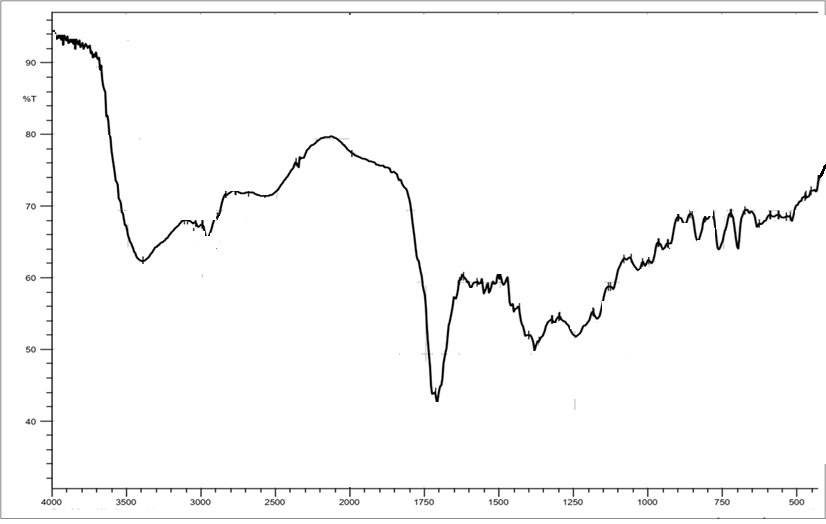
 Fig S6 IR spectrum of SOL IMP A and SOL IMP E


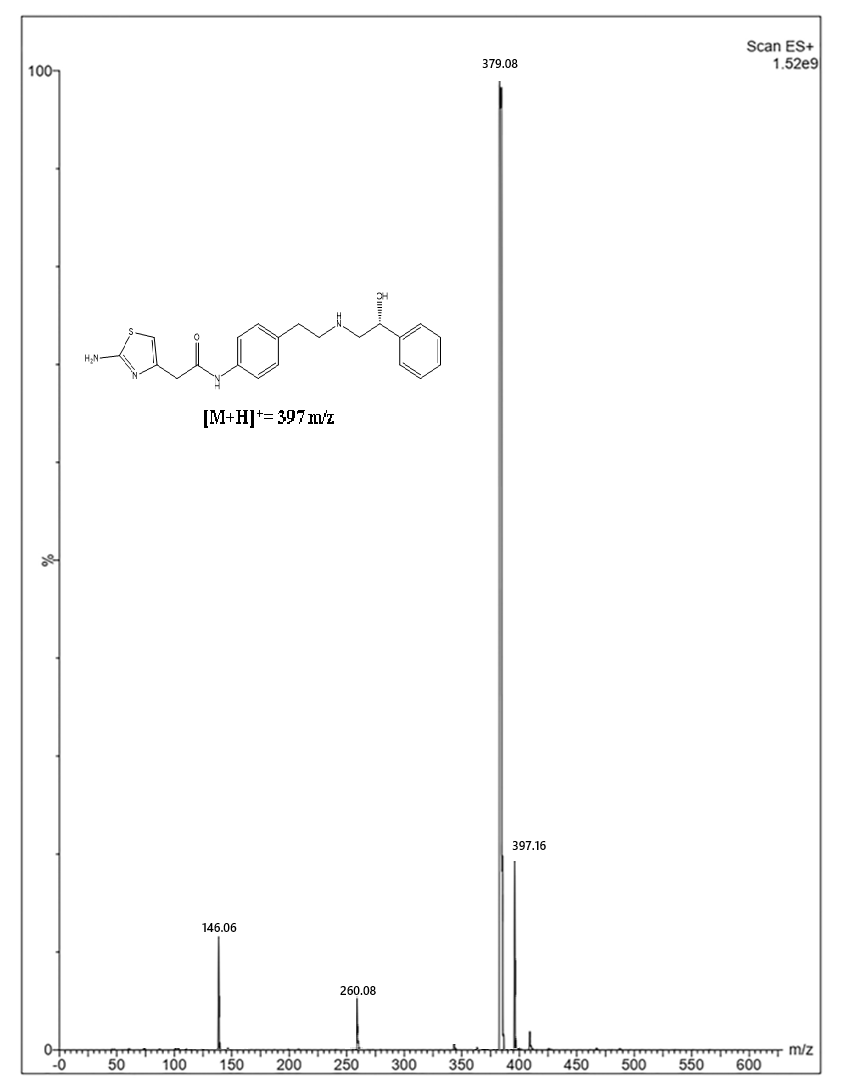


Fig S7 MS spectrum of MIR


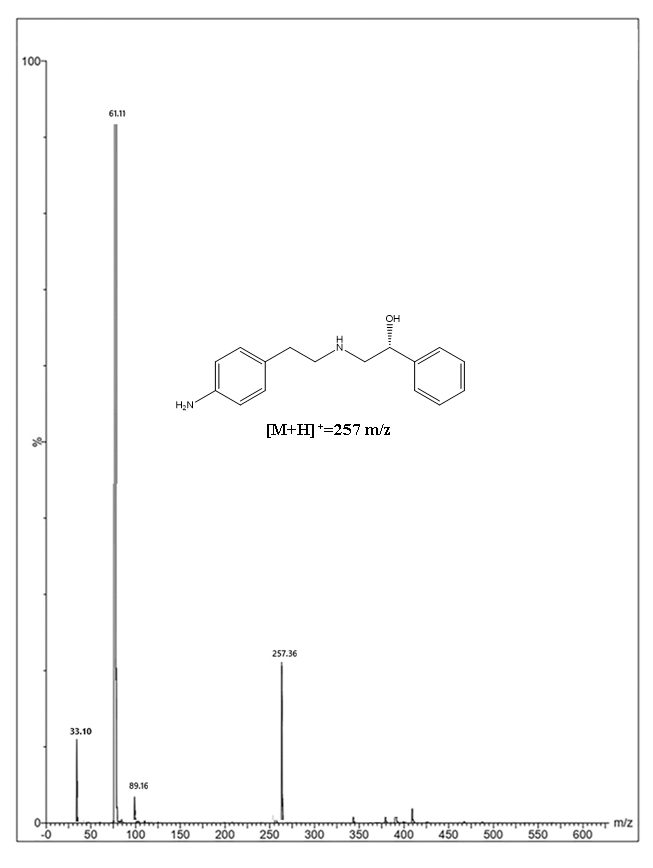


Fig S8 MS spectrum of MIR MET


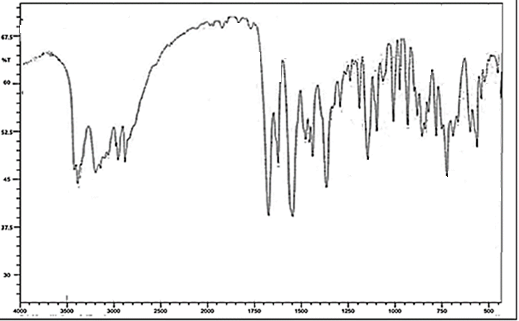


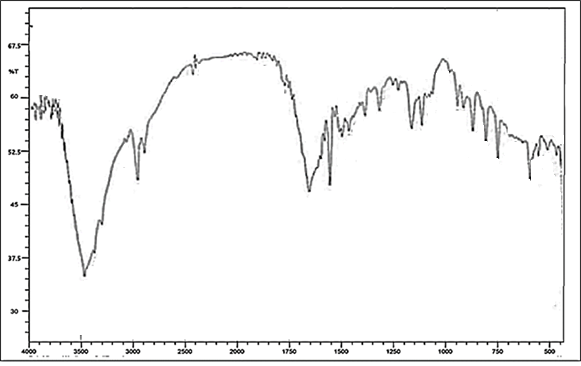
Fig S9 IR spectrum of MIR

Fig S10 IR spectrum of MIR MET

Table S1: Comparison of the proposed HPLC method with previously reported methods for the determination of solifenacin and mirabegron

| Reference | Analytes determined | Technique | Linearity range | LOD | LOQ | Run time (min) | Remarks |
| --- | --- | --- | --- | --- | --- | --- | --- |
| Shah et al., 2019 [12] | SOL + MIR | HPTLC | SOL - MIR: 4–1.1 µg /band-2–5.5 µg/ band0 | Sol 0.93  MIR0.3 | Sol 2.88  MIR0.93 | — | Lower sensitivity, not stability-indicating |
| Andhale et al., 2022 [43] | SOL + MIR | RP-HPLC | SOL & MIR: 5–50 µg/mL | Sol 1.3  MIR1.11 | Sol 3.87  MIR3.43 | ~25 | No impurity or metabolite analysis |
| Kadam et al., 2023 [44] | SOL + MIR | UHPLC | SOL & MIR: 1–50 µg/mL | Sol 0.23  MIR0.1 | Sol 0.65  MIR0.37 | ~18 | High efficiency, no impurity/metabolite profiling |
| Nour et al., 2024 [45] | SOL + MIR | Spectrofluorimetry | SOL - MIR:5–250 and 50–600 ng/mL | Sol 1.2  MIR10.23 | Sol 3.54  MIR30.43 | — | High sensitivity, lacks chromatographic separation or stability studies |
| Bairam et al., 2024 [47] | SOL + MIR | RP-HPLC (AQbD) | SOL - MIR: 5-25- 25- 125 µg/mL | Sol 1,23  MIR5.34 | Sol 3.43  MIR16.32 | ~22 | AQbD-based, no degradation products studied |
| Proposed method | SOL, MIR, SOL IMP A, I, E, MIR MET | RP-HPLC | SOL & MIR: 1–100 µg/mL | Sol 0.295  MIR0.32 | Sol 0.89  MIR0.99 | 10.5 | Simultaneous stability-indicating determination of drugs, impurities, and metabolites with short run time and high sensitivity |
